# Characterization of Staufen1 Ribonucleoproteins by Mass Spectrometry and Biochemical Analyses Reveal the Presence of Diverse Host Proteins Associated with Human Immunodeficiency Virus Type 1

**DOI:** 10.3389/fmicb.2012.00367

**Published:** 2012-10-25

**Authors:** Miroslav P. Milev, Mukunthan Ravichandran, Morgan F. Khan, David C. Schriemer, Andrew J. Mouland

**Affiliations:** ^1^HIV-1 Trafficking Laboratory, Lady Davis Institute at the Jewish General HospitalMontréal, QC, Canada; ^2^Division of Experimental Medicine, Department of Medicine, McGill UniversityMontreal, QC, Canada; ^3^Department of Biochemistry and Molecular Biology, University of CalgaryCalgary, AB, Canada; ^4^Department of Microbiology and Immunology, McGill UniversityMontreal, QC, Canada

**Keywords:** Gag, genomic RNA, HIV-1, mass spectrometry, gradient centrifugation, ribonucleoprotein, Staufen1, virus-host interactions

## Abstract

The human immunodeficiency virus type 1 (HIV-1) unspliced, 9 kb genomic RNA (vRNA) is exported from the nucleus for the synthesis of viral structural proteins and enzymes (Gag and Gag/Pol) and is then transported to sites of virus assembly where it is packaged into progeny virions. vRNA co-exists in the cytoplasm in the context of the HIV-1 ribonucleoprotein (RNP) that is currently defined by the presence of Gag and several host proteins including the double-stranded RNA-binding protein, Staufen1. In this study we isolated Staufen1 RNP complexes derived from HIV-1-expressing cells using tandem affinity purification and have identified multiple host protein components by mass spectrometry. Four viral proteins, including Gag, Gag/Pol, Env and Nef as well as >200 host proteins were identified in these RNPs. Moreover, HIV-1 induces both qualitative and quantitative differences in host protein content in these RNPs. 22% of Staufen1-associated factors are virion-associated suggesting that the RNP could be a vehicle to achieve this. In addition, we provide evidence on how HIV-1 modulates the composition of cytoplasmic Staufen1 RNPs. Biochemical fractionation by density gradient analyses revealed new facets on the assembly of Staufen1 RNPs. The assembly of dense Staufen1 RNPs that contain Gag and several host proteins were found to be entirely RNA-dependent but their assembly appeared to be independent of Gag expression. Gag-containing complexes fractionated into a lighter and another, more dense pool. Lastly, Staufen1 depletion studies demonstrated that the previously characterized Staufen1 HIV-1-dependent RNPs are most likely aggregates of smaller RNPs that accumulate at juxtanuclear domains. The molecular characterization of Staufen1 HIV-1 RNPs will offer important information on virus-host cell interactions and on the elucidation of the function of these RNPs for the transport of Gag and the fate of the unspliced vRNA in HIV-1-producing cells.

## Introduction

HIV-1 infection is characterized by a progressive depletion of CD4 + T lymphocytes that makes patients susceptible to opportunistic diseases and ultimately leads to the development of acquired immunodeficiency syndrome (AIDS; Ho et al., 1995; Lindwasser et al., 2007). HIV-1 replication is divided into early and late events (Wang et al., 2000; Freed, 2001): the early events include virus entry, uncoating of the viral core that contains the vRNA, reverse transcription of vRNA to cDNA and finally, the integration of the resulting viral double-stranded DNA into host chromosomes. The later events include the transcription of the proviral genome to generate a primary transcript, the vRNA, its processing, maturation and nucleocytoplasmic export and also the synthesis of viral structural proteins, virus assembly and budding. Following transcription, the vRNA either remains unspliced or is spliced to generate more than 30 distinct mRNAs that are grouped into singly spliced, 4 kb mRNAs (encoding the auxiliary proteins Vif, Vpr, Vpu and the glycoprotein, Env) or into multiply spliced, 1.8 kb mRNA species (encoding the early viral regulatory proteins Tat, Rev and Nef; Arrigo et al., 1990; Schwartz et al., 1990; Purcell and Martin, 1993). The 1.8 kb RNAs are constitutively exported from the nucleus early following transcription, while the nuclear export of vRNA and the 4 kb species is dependent on the CRM1/Exportin1 export pathway (Yi et al., 2002). These events are well orchestrated, dynamic and depend on the activities of viral as well as select host cell proteins and machineries that are co-opted by the virus.

The two largest viral mRNA species, which contain intronic sequences, are both exported from the nucleus and translated in the cytoplasm. While usually mRNAs with introns are tagged as aberrant since they are “incompletely spliced” and are degraded by cellular RNA quality control machineries (Doma and Parker, 2007), these viral mRNAs are quite stable (Mouland et al., 2002), and therefore likely evade this surveillance machinery by co-opting host proteins involved in this process (Ajamian et al., 2008; Nathans et al., 2009). Furthermore, vRNA has an additional fate in that it can also be packaged into progeny virions (Butsch and Boris-Lawrie, 2002). This latter step is made possible by a selective interaction between vRNA and its gene product, the precursor Group specific antigen, pr55^Gag^ (termed Gag herein). Gag interacts with a packaging signal in the 5′UTR of vRNA for selection into assembling virions (Lever et al., 1989; Clever et al., 1995). Like other mRNAs, vRNA is likely transported through the cytoplasm in the context of an RNP (Wilhelm and Vale, 1993; Mouland et al., 2001; Lehmann et al., 2009). Indeed, mRNA-binding proteins such as Staufen1 associate closely with Gag to form the HIV-1 RNP that also incorporates vRNA, but none of the spliced HIV-1 RNAs (Chatel-Chaix et al., 2004; Cochrane et al., 2006). Moreover, recent work has demonstrated that HIV-1 RNPs that contain Staufen1 take advantage of endosomal machineries for intracellular trafficking (Lehmann et al., 2009; Molle et al., 2009). Nevertheless, these later events still remain one of the most understudied areas of HIV-1 biology.

Generally, like other cellular RNPs, the composition of cytosolic HIV-1 RNPs is plastic in nature such that proteins may engage in the nucleus, disengage and/or be acquired during transit from the nucleus to the cytoplasm and during the assembly of vRNA into viral particles. The composition of the HIV-1 RNP has not been completely characterized, however. Recent work supports the idea that vRNA interacts with Gag at juxtanuclear and cytoplasmic domains (Poole et al., 2005; Levesque et al., 2006) and considerable efforts are now being made to evaluate how HIV-1 co-opts factors following the nuclear export of the vRNA – a late step in HIV-1 replication that includes RNA transport, utilization (translation) and degradation (Cochrane et al., 2006; Lehmann et al., 2009; Molle et al., 2009; Kemler et al., 2010). The formation of the HIV-1 RNP is initially achieved by the binding of Gag via its nucleocapsid (NC) domain and the RNA packaging signal *psi* in the 5′-end of the vRNA. This early capture may govern the directed movement of vRNA along the cytoskeleton, to the translation apparatus, to sites of viral assembly and finally, into assembling viral particles. A number of host gene products, such as hnRNP A1, PSF/nsr54 and APOBEC3G associate with vRNA (Beriault et al., 2004; Khan et al., 2005). Furthermore, a limited set of host *trans*-acting proteins mediates trafficking by binding to specific *cis*-acting sequence elements in vRNA (Mouland et al., 2001; Levesque et al., 2006). While the stability and functionality of HIV-1 RNP is most probably a result of interactions between a few viral (i.e., Gag) and host cell proteins such as Staufen1 and Upf1 (Up-frameshift protein 1; Chatel-Chaix et al., 2004; Ajamian et al., 2008), the molecular composition of the HIV-1 RNP likely changes and is dictated by HIV-1, either by direct recruitment of, or by binding to host cell factors.

Staufen1 belongs to a growing family of the double-stranded RNA-binding proteins (dsRBPs) that includes protein kinase dsRNA dependent (PKR), the activator of PKR (PACT), TAR-RNA binding protein (TRBP) and RNA Helicase A (RHA, reviewed in (Fierro-Monti and Mathews, 2000; Saunders and Barber, 2003; Tian et al., 2004) and is a principal component of various RNPs that are engaged in the localization and trafficking of cellular mRNAs (Kiebler et al., 1999; Kiebler and DesGroseillers, 2000; Roegiers and Jan, 2000). Staufen1-containing high-molecular weight complexes ranging in size from 10–30 MDa appear as granules dispersed in the cytoplasm of eukaryotic cells. Several components are found in these complexes such as ribosomes, tubulin, actin, dynein, RHA, hnRNP U and nucleolin (Brendel et al., 2004; Villace et al., 2004). RNA-binding by Staufen1 regulates diverse classes of mammalian mRNAs that encode proteins with functions in different metabolic pathways and cellular physiological processes (Kim et al., 2007; Furic et al., 2008).

Our previous work demonstrated that Staufen1 binds to both Gag as well as its vRNA substrate (while excluding all of the spliced HIV-1 RNA species), which likely drives the incorporation of Gag and vRNA into assembling virions through the formation of a HIV-1 RNP (Mouland et al., 2000; Chatel-Chaix et al., 2004). Furthermore, modulating the levels of Staufen1, by siRNAs and overexpression, perturbs HIV-1 assembly, including Gag multimerization and vRNA encapsidation, resulting in negative effects on viral infectivity (Mouland et al., 2000; Chatel-Chaix et al., 2004, 2007, 2008; Abrahamyan et al., 2010). Staufen1 also influences the anterograde trafficking of Gag. Both Staufen1 and Gag were shown to associate in the cytoplasm and also at cholesterol-enriched lipid rafts, which are virus assembly domains (Milev et al., 2010).

Staufen1 potentially may play similar roles in the replication of other retroviruses, such as, HIV-2 and MLV since it was found incorporated within them (Mouland et al., 2000). Importantly it does not associate with any tested DNA virus, including adenovirus, Epstein-Barr virus and human herpesvirus 6, supporting its preferential role in the biology of RNA viruses. In a yeast two-hybrid screen and in co-immunoprecipitation (IP) experiments, Staufen1 was identified as an interactive partner of the influenza A virus non-structural protein, NS1 (Falcon et al., 1999). Recent work demonstrated that Staufen1 also associates to viral RNPs and viral RNAs in influenza virus-infected cells (de Lucas et al., 2010). Staufen1 also associates with the 3′UTR of HCV RNA, the sequence essential for the initiation of (−) strand synthesis (Harris et al., 2006). Together with the numerous other cellular proteins found to interact with this region, Staufen1 most probably also plays a role in HCV replication.

The aim of the work described here was to examine the composition of the Staufen1 RNP proteome and how it is modulated by HIV-1. To do this, Staufen1-binding proteins were purified using tandem affinity purification (TAP) in HIV-1-expressing cells and identified by mass spectrometry. Approximately 200 host proteins were identified using this strategy. The Staufen1 HIV-1 RNP that bound precursor Gag shared many proteins with cytosolic RNA trafficking RNPs. However, notable compositional differences of the Staufen1 RNPs were induced by HIV-1. Furthermore, ∼22% of the identified proteins are found in isolated HIV-1 particles. Biochemical and imaging analyses confirmed many of these associations. Biochemical fractionation of cellular RNPs revealed further characteristics of the Staufen1 RNPs that are assembled in HIV-1-producing cells. Our results provide a comprehensive view on the composition of Staufen1-containing HIV-1 RNPs and their functional importance that likely resides in the fate of the vRNA.

## Materials and Methods

### Cells and cell lines

Human embryonic kidney 293T cells HeLa, and Jurkat T cells were maintained at 37°C in Dulbecco’s modified Eagle’s or RPMI-1640 medium supplemented with 10% decomplemented fetal bovine serum (FBS) and 100 U/ml penicillin, 100 mg/ml streptomycin (Invitrogen). Stable TAP and Staufen1-TAP neomycin-resistant cell lines were generated in 293T and Jurkat T cells. Briefly, 293T cell were plated in 60 mm dishes and Jurkat T cells in 25 cm^2^ flasks. The following day these were transfected with 2 and 4 μg plasmid DNA, respectively, using Lipofectamine 2000 according to the protocol provided by the manufacturer (Invitrogen). 24 h post-transfection, 600 μg/ml G418 (Invitrogen) was added to the medium for selection. The cell cultures were maintained at 90% confluence and subsequently, sub-cultured at lower densities. Resistant clones were isolated 14 days following selection. Tissue culture medium was removed from the plates, and cells were washed with sterile PBS. To pick colonies, sterile 3 mm cloning disks were dipped in trypsin solution and placed on colonies for 30 s. The colonies adhered to the cloning disks and were transferred to 24-well dishes. When the cells reached pre-confluence, they were transferred to 6-well plates. For suspension Jurkat T cells, the procedure for isolation of stable clones differed as follows: cells were maintained in RPMI-1640 with 600 μg/ml G418 for 14 days and then were serially diluted and transferred in 24-well dishes for expansion. The surviving stable lines exhibited various expression levels of the TAP tagged Staufen1 as assessed by SDS-PAGE and western blotting. Staufen1-TAP expression levels were constant for each clone and were stable for at least 20 subsequent passages.

### Transient transfections

For affinity purification experiments, transfection of control TAP and Staufen1-TAP stable cell lines with proviral DNA, pNL4-3, was carried out in 150 cm^2^ flasks (Nunc). The cells were plated at 5 × 10^6^ per flask for 24 h before transfection. 20 μg of plasmid and 50 μl Lipofectamine per flask were added, cells were incubated for 20 min and then mixed with tissue culture medium. For IP analyses, HeLa cells were transfected with corresponding amounts of plasmid DNA and cell lysates were prepared as described previously (Mouland et al., 2000; Chatel-Chaix et al., 2004). The overexpression of IMP1 (in the context of IMP1-VenC fusion protein) was performed in both HeLa and 293T cells. Transfection efficiencies were greater than 65% in all experiments (range 65–80%).

Staufen1-HA, pNL4-3 and pNL4-XX and transfection of HeLa cells were described previously (Mouland et al., 2000; Chatel-Chaix et al., 2004; Ajamian et al., 2008). For sucrose gradient fractionation experiments, transfection of proviral DNA, pNL4-3 was carried out in 75 cm^2^ flasks (Nunc). The cells were plated at 3 × 10^6^ per flask for 12 h before transfection. Transfection efficiencies were greater than 70% in all experiments (range 65–80%). For RNase A treatments, cell lysates were incubated for 30 min at 4°C with RNase T1 at 1 U/mL. HeLa cells were transfected with either non-silencing siRNA (siNS) or Staufen1 siRNA (siStaufen1) at a final concentration of 10 nM. siRNA transfections were performed with lipofectamine 2000. Thirty-six hours later, the cells were washed with ice cold PBS and lysed in XB buffer for subcellular fractionation in sucrose gradients (see below; Chatel-Chaix et al., 2004).

### Tandem affinity purification and western blotting

The purification of Staufen1 complexes was adapted using the TAP protocols as described previously (Puig et al., 2001; Villace et al., 2004). Briefly, after transfection, cells were lysed in buffer (50 mM Tris-HCl pH 7.5, 5 mM EDTA, 100 mM NaCl, 1 mM DTT, 0.5% NP-40) and complete protease inhibitor cocktail (Roche). Cell extracts were centrifuged at 4°C for 5 min at 5000 rpm and the recovered supernatants were centrifuged for 15 min at 14,000 × *g*. The lysates were stored at −20°C. In the first affinity purification step, 20–75 mg cell lysates were applied to IgG Sepharose six Fast Flow beads (Amersham Biosciences; at a ratio of 5 μl beads per 1 mg protein). The resin was prepared according to the manufacturer’s protocol. After overnight incubation at 4°C, the IgG resin was washed 10 times with 10 volumes of IPP-150 buffer (10 mM Tris-HCl pH 8, 150 mM NaCl, 0.1% NP-40) and five times with 10 volumes of TEV cleavage buffer – TCB (10 mM Tris-HCl pH 8, 150 mM NaCl, 0.1% NP-40, 0.5 mM EDTA). 20 U of ActivTEV – Tobacco Etch Virus protease (Invitrogen, Carlsbad, USA) in TCB (300 μl) was then added to the resin and rotated for 2 h at room temperature in order to release the complexes bound to the resin (100 μl). In the second affinity purification step, the supernatant after TEV cleavage was adjusted with CaCl_2_ to a 2 mM final concentration and incubated overnight with 60 μl Stratagene Calmodulin-affinity resin (Agilent Technologies, Cedar Creek, USA). The resin was then washed three times with 10 volumes of Calmodulin-binding buffer, CBB (10 mM beta-mercaptoethanol, 10 mM Tris-HCl pH 8, 150 mM NaCl, 0.1% NP-40, 1 mM imidazol, 1 mM MgOAc, 2 mM CaCl_2_) and two times with 10 volumes of Calmodulin-rinsing buffer, CRB (50 mM ammonium bicarbonate pH 8.0, 75 mM NaCl, 1 mM MgOAc, 1 mM imidazol, 2 mM CaCl_2_). For the elution of complexes, the beads were resuspended and incubated for several minutes with ∼100 μl of Calmodulin-elution buffer, CEB (50 mM ammonium bicarbonate, 15–25 mM EGTA). Western blotting of input cell lysates and affinity-purified eluates was performed by standard procedures (Abrahamyan et al., 2010) using several of the primary antisera described below. TAP and western blotting results are representative of >5 independent experiments.

### Coomassie blue staining and gel slice excision

For mass spectrometry analysis, the eluates were fractionated on 4–12% SDS-PAGE and the gels were then subjected to three 5 min washes in 300 ml double distillated water (ddH_2_O) and stained with 100 ml Bio-Safe Coomassie stain (Bio-Rad) for 60 min followed by destaining. In total, 23 gel slices from each experiment (St-TAP or St-TAP + HIV-1) were excised, placed in a pre-washed, low-retention 1.5 ml snap-cap tubes for subsequent in gel digestion and liquid chromatography and mass spectrometry (LC-MS) analysis.

### In gel destaining and digestion

Gel bands were diced into ∼1 mm^2^ pieces and rinsed once with 200 μl HPLC-grade water, twice with 200 μl 25 mM ammonium bicarbonate in 50% (v/v) acetonitrile, followed by 100 μl acetonitrile to dehydrate the gel plugs, which were then lyophilized. The dry gel plugs were rehydrated in 5–7 μl of 25 mM ammoniumbicarbonate, pH 8.0, containing 12.5 ng/μl trypsin. After rehydration, an additional 30 μl of 25 mM ammonium bicarbonate was added and the gel plugs were incubated overnight at 37°C. Peptides were extracted from gel plugs by two rounds of incubation with 50 μl of 1% formic acid in 50% acetonitrile. The pooled extracts were reduced to dryness and reconstituted in mobile phase A for reversed phase chromatography.

### Mass spectrometry

Digests were analyzed using an Agilent 1100 LC-Ion-Trap-XCT-Ultra system (Agilent Technologies, Santa Clara, CA, USA) fitted with an integrated fluidics cartridge for peptide capture, separation, and nano-spraying (HPLC Chip). Injected samples were trapped and desalted on a pre-column channel (40 nl volume; Zorbax 300SB-C_18_) for 5 min with mobile phase A (3% acetonitrile, 0.2% formic acid) delivered by an auxiliary pump at 4 μl/min. The peptides were then reverse-eluted from the trapping column and separated on the analytical column (150 mm length; Zorbax 300SB-C_18_) at 0.3 μl/min. Peptides were eluted using a 5–70% gradient in mobile phase B (97% acetonitrile, 0.2% formic acid) over 45 min. MS/MS spectra were collected by data-dependent acquisition, with parent ion scans of 8,100 Th/s over *m*/*z* 300–2,000 and MS/MS scans at the same rate over *m*/*z* 100–2,200. Peak-list data were extracted from these files by DataAnalysis software for the 6300 series ion trap, v3.4 (build 175). Mascot v2.2 (MatrixScience, Boston, MA, USA) was used to search the MS/MS data using the following parameters: 1.6 Da precursor ion mass tolerance, 0.8 Da fragment ion mass tolerance, one potential missed cleavage, and oxidized methionine as a variable modification. NCBI nr 2008.01.03 (5,824,077 sequences) was searched, with a restriction to “other viruses” and humans. Mass spectrometry analyses on isolated TAP eluates were reproduced at least two times in independent experiments.

### Antibodies and reagents

Mouse anti-Staufen1, anti-UPF (1, 2 and 3), anti-RHA and anti-AUF1 antibodies were provided by Luc DesGroseillers (Université de Montréal), Jens-Lykke Andersen (University of California), Juan Ortin (Centro Nacional de Biotecnologia, Madrid, Spain) and William Rigby (Dartmouth University, NH, USA), respectively (Villace et al., 2004; Ajamian et al., 2008; Abrahamyan et al., 2010). Rabbit anti-IMP1 and anti-ABCE1 antibodies were generous gifts from Finn Nielsen (University of Copenhagen) and from Jaisri Lingappa (University of Washington; Zimmerman et al., 2002; Milev et al., 2010), respectively. Mouse anti-Tsg101 and anti-L7 antibodies were purchased from Novus Biologicals (Littleton, USA), anti-eF1α, anti-TDP-43 and anti-CRM1 were purchased from Upstate (Millipore), ProteinTech Group (Chicago, USA) and Santa Cruz (California, USA), respectively; rabbit anti-p17 and sheep anti-gp120 were obtained from the NIH (Abrahamyan et al., 2010). For all immunofluorescence (IF) experiments, secondary AlexaFluor anti-mouse, anti-rabbit or anti-sheep conjugated antibodies were used (Invitrogen) as previously described (Milev et al., 2010).

### Immunofluorescence, FISH and confocal microscopy

At 24–48 h after transfection, cells were washed two times with 1× PBS and fixed in 4% PFA for 20 min, washed two times with 1× PBS and treated with 0.2% Triton X-100 for 10 min. IF and FISH were performed essentially as previously described (Lehmann et al., 2009; Milev et al., 2010; Vyboh et al., 2012). Cells were then incubated for 10 min with 0.1 M glycine, washed and blocked for 30 min in 1× BSA (Roche Applied Science, Germany). The primary antibody was incubated for 1.5 h at room temperature. Cells were washed 2× with PBS and subsequently incubated with secondary antibody for 30 min. After this step the glass coverslips were washed 2× with PBS, mounted on slides and visualized with a Carl Zeiss Pascal LSM5 laser scanning confocal microscope (Carl Zeiss, Germany).

### Expression vectors

Plasmids pcDNASt-TAP and pcDNA-TAP were constructed as previously described (Villace et al., 2004). Proviral DNA, pNL4-3 was described in previous work (Adachi et al., 1986; Chatel-Chaix et al., 2004). pNL4-XX, a proviral DNA derived from pNL4-3, harbors two mutations in the *gag* open reading frame to prevent Gag and Gag/Pol synthesis, was described elsewhere (Poon et al., 2002; Abrahamyan et al., 2010). Constructs expressing Staufen1-HA, pGL3-IMP1-VenusC and pGL3-MS2-Venus (full length) were described previously (Milev et al., 2010). pCMV-Gag-RRE was described earlier (Lingappa et al., 2006).

### Subcellular fractionation

Following transfection, cells were washed with ice cold PBS, homogenized in an equal volume of XB Buffer (20 mM HEPES pH 7.9, 1.5 mM MgCl_2_, 0.5 mM DTT, protease inhibitor cocktail). The homogenate was centrifuged for 10 min at 5,000 rpm to remove the insoluble material. The supernatant (1 mg total protein) was applied on the top of a 5 ml 5–50% pre-loaded sucrose gradient in XB buffer, as described (Yoon and Mowry, 2004). Lysates were separated by centrifugation at 44,000 rpm for 2 h in SW55i beckman rotor at 17°C. 18–20 250 μl fractions were collected and resolved by SDS-PAGE or processed for slot blot analyses.

### RNA slot blot analyses

In order to evaluate vRNA fractionation in sucrose gradient fractions, an aliquot of each eluate was mixed 1:3 with GTC and FFM solution (10× OPS buffer, 37% Formaldehyde, 99% Formamide) and heated at 65°C for 15 min and transferred to 0.45 μm nylon membrane by intermittent suction for slot blot analyses using a Gibco/BRL slot blot apparatus. A ^[32]^P-labeled cDNA probe to the HIV-1 5′ *gag* coding region to recognize the vRNA only was prepared using random prime labeling kit, as described (Yao et al., 1998; Mouland et al., 2000).

## Results

### Generation of Staufen1-TAP and control TAP stable cell lines

**Figure 1:**
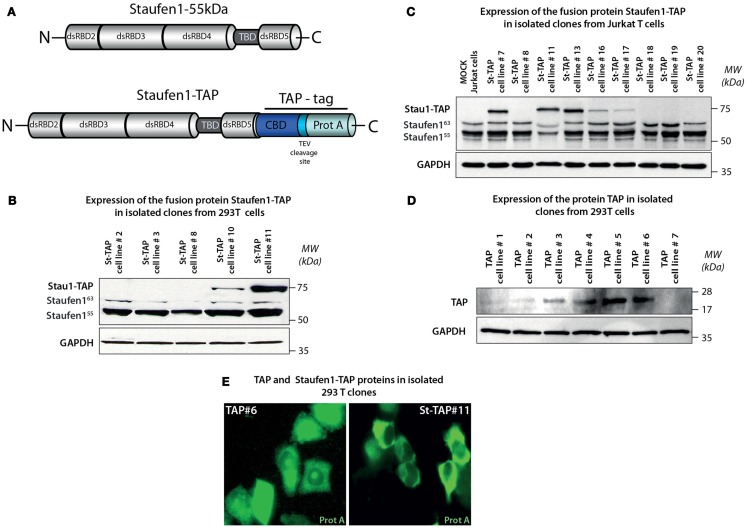
**Generation and isolation of stable neomycin-resistant cell lines expressing Staufen1-TAP and TAP proteins**. **(A)** Structures of the Tandem Affinity Purification (TAP) tag cassette and Staufen1-TAP fusion protein. TAP tag consists of two sequences responsible for the affinity purification – IgG binding domain of Protein A and Calmodulin-binding peptide separated by a unique cleavage site for Tobacco Etch Virus (TEV) protease. **(B,C)** Expression levels of St-TAP in stable 293T and Jurkat T cell clones detected by western blotting analysis using a monoclonal mouse anti-Staufen1 antibody that recognizes both Staufen1^55^ (55 kDa), Staufen1^63^ (63 kDa), the endogenous isoforms and exogenous St-TAP (∼75 kDa) fusion protein, respectively. GAPDH is the loading control. 293T cell clone #11 and Jurkat T cell clone #13 were used in experiments. **(D)** Expression levels of TAP protein in 7 (#1–#7) control 293T clones and verified by western blotting. Clone #6 was used in control experiments. **(E)** Stable cell lines TAP#6 and St-TAP#11 were stained with mouse monoclonal anti-protein A antibody. The primary antibody was detected by Alexa Fluor 488 goat anti–mouse IgG antibody and the stained cells were visualized by epifluorescence microscopy.

Tandem affinity purification is a powerful method for the specific isolation of protein complexes under native conditions. In order to characterize Staufen1 HIV-1 RNP complexes and to determine Staufen1-binding partners, we generated neomycin-resistant human 293T and Jurkat T cell lines expressing Staufen1-TAP (or St-TAP) and TAP control cell lines (Figures 1A–D; Puig et al., 2001). Cells were transfected with pcDNA3 in which a TAP tag was cloned at the carboxy-terminus of the Staufen1^55 kDa^ cDNA (Villace et al., 2004; Ajamian et al., 2008). Previous studies have demonstrated that St-TAP protein has the same properties as the native protein (Villace et al., 2004). Two of the 12 single-cell clones that were expanded in expressed the fusion protein as assessed by western blotting. St-TAP#11 was used for all subsequent experiments (Figure 1B; shown in Ajamian et al., 2008), because the expression levels were similar to that of endogenous Staufen1^55 kDa^. Jurkat St-TAP#13 (Figure 1C) was used for subsequent purification and characterization of Staufen1 complexes. A control cell line was also generated expressing only the TAP tag (Figure 1D). The subcellular distribution of TAP and St-TAP proteins in these stable 293T cell lines were assessed by IF using a monoclonal anti-Protein A antibody that recognizes the IgG binding domain of Protein A (the carboxy-terminal part of the TAP tag). TAP tag was found to be uniformly distributed throughout the cell (Figure 1E, left panel) and St-TAP was found principally in the cytoplasm of stable expressing cells (Figure 1E, right panel), corresponding to the localization pattern of endogenous Staufen1 (Wickham et al., 1999; Thomas et al., 2005). These results indicate that the Staufen1 component of the fusion protein, rather than the TAP tag, is responsible for correctly localizing the fusion protein to the cytosol.

### Purification of Staufen1 HIV-1 ribonucleoprotein complexes

**Figure 2:**
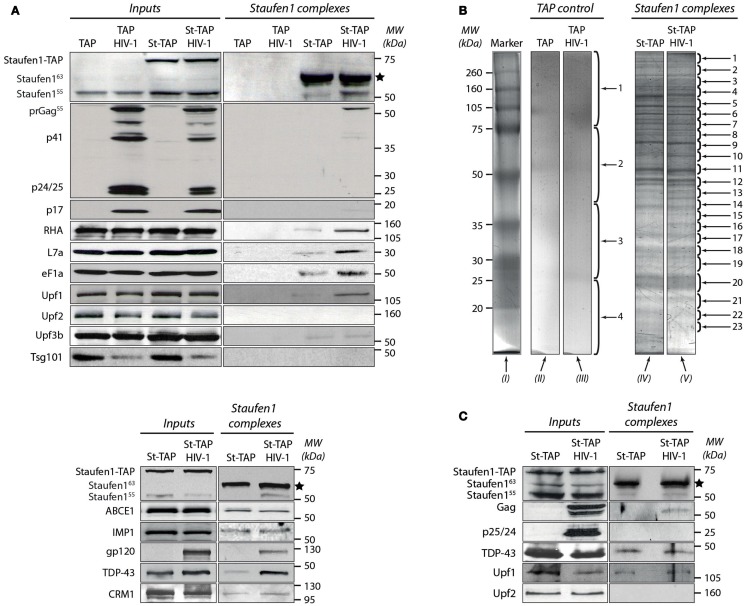
**Characterization of the tandem affinity-isolated Staufen1^55 kDa^ RNPs by western blot and mass spectrometry analysis**. **(A)** Separation of proteins from cell lysates (*Inputs*) before (left panels) and after (right panels) tandem affinity purification (*Staufen1 complexes*) 0.25 mg of total protein obtained from extracts of TAP (control) or Staufen1-TAP expressing cell lines in the absence or presence of HIV-1 were used for affinity purification. Initially, the membrane was blotted with mouse anti-Staufen1 monoclonal antibody shown on the top left and right panels. The star (*) indicates the position of the purified St-CBP protein. **(B)** The proteins from the eluted Staufen1^55 kDa^ complexes and those from TAP alone (control) were purified from 75 mg of total protein, separated on SDS-PAGE and subsequently stained with Bio-Safe Coomassie Blue. Four bands were excised from gel lanes *(II)* and *(III)* for the identification of potential contaminating proteins. 23 bands from St-TAP and St-TAP HIV-1 (lanes *IV* and *V)* were excised and analyzed with mass spectrometry. Lane *I* represents the molecular weights in kDa of the protein standards. **(C)** Dual affinity purification of Staufen1 complexes derived from Jurkat T St-TAP stable cell lines in the absence or in the presence of HIV-1.

We used the TAP-to-MS protocol to discover compositional changes in Staufen1-containing complexes when HIV-1 is expressed. We performed parallel purifications of extracts obtained from stably expressing St-TAP (Mock) or TAP (control) cell lines transiently transfected with pNL4-3. At 40 h post-transfection cells were harvested and 20 mg total protein was used for TAP purification. We also isolated Staufen1 complexes from both HIV-1 infected or uninfected Jurkat T cells that expressed St-TAP. Staufen1^55 kDa^ and St-TAP were present in RNPs both before and after affinity purification (Figures 2A,C). The TEV protease-mediated cleavage of St-TAP (75 kDa) fusion proteins resulted in the formation of St-CBP protein that migrated at ∼64 kDa (Figures 2A,C). Both endogenous 55 and 63 kDa isoforms of Staufen1 were recruited to RNPs likely due to their ability to form homo- and heterodimers and to accumulate in RNPs (Martel et al., 2010). When HIV-1 was expressed, higher amounts of endogenous Staufen1 were found in the eluates. Furthermore, the precursor Gag protein was found in association with Staufen1 but not any of its smaller, mature cleavage products [CA (p24), MA (p17), NC (p7), or p6 (Figures 2A,C)]. This result is in accordance with our previous data showing the selective manner in which Staufen1 and Gag interact (Chatel-Chaix et al., 2004). Interestingly, in the majority of the experiments we also detected pr160^Gag/Pol^ (Gag/Pol), probably as a result of its interaction with Staufen1, Gag and the presence of vRNA that facilitates such associations during HIV-1 assembly (Khorchid et al., 2002). The affinity purification was validated by western blotting using antibodies against several proteins that were previously found to associate with Staufen1 such as RHA, ribosomal protein L7a (Villace et al., 2004) and poly-A binding protein (PABP; Figure 2A; Miroslav P. Milev and Andrew J. Mouland, data not shown). Western blotting analysis revealed that eukaryotic translation elongation factor-1α (eF1α) is a Staufen1-interacting partner. This is the first time that the association with eF1α with Staufen1 has been reported; it nevertheless interacts with Gag and is incorporated in HIV-1 particles (Cimarelli and Luban, 1999).

Previously, Staufen1 was found to interact with the nucleocytoplasmic shuttling protein, Barentsz (Macchi et al., 2003), a component of exon junction complexes and an important player in nonsense-mediated mRNA decay (NMD) – a surveillance process that degrades aberrant mRNAs containing premature termination codons (Palacios et al., 2004). Staufen1-mediated mRNA decay was also described to involve Staufen1 and the major NMD factor, Upf1 (Kim et al., 2005). Moreover, these two proteins are found in association with APOBEC3G RNPs that might be involved in retroviral restriction (Kozak et al., 2006). These findings provide molecular and biochemical links between mRNA splicing, trafficking and decay. Therefore we wanted to determine the potential association of some of the main NMD factors such as Upf1, Upf2, and Upf3 in Staufen1-containing RNPs in the absence or presence of HIV-1 infection. Our results clearly demonstrate the association of Upf1 with the Staufen1 HIV-1 RNP complexes. When HIV-1 was expressed, we consistently observed approximately threefold more Upf1 eluting from the Staufen1 column compared to that found when HIV-1 was not expressed (Figures 2A,C). Upf2 was not detected in any of the experiments using 293T or Jurkat T Staufen1-TAP cell lines (Figures 2A,C) indicating that Upf2 is absent or is not stably bound in the Staufen1 RNP (Ajamian et al., 2008). The absence of Upf2 was expected since Staufen1 and Upf2 compete for binding with Upf1. Upf3b was also detected in St-TAP complexes derived from 293T cells as assessed by western blotting (Figure 2A).

We have demonstrated an important role for Staufen1 in the process of Gag multimerization, trafficking and viral assembly that could be coordinated with a role in the encapsidation of vRNA (Chatel-Chaix et al., 2007; Abrahamyan et al., 2010; Milev et al., 2010). We wished to verify whether the function of Staufen1 in Gag multimerization, assembly and vRNA encapsidation are linked to the function of other cellular Gag-interacting factors. To this end, we chose the host ATP-binding cassette protein ABCE1 which associates with Gag shortly after its synthesis (Dooher et al., 2007) and is critical for the proper generation of an immature HIV-1 capsid (Zimmerman et al., 2002). As in seen with Staufen1, the NC domain of Gag is a necessary and sufficient determinant for binding ABCE1 (Zimmerman et al., 2002; Lingappa et al., 2006). Equal amounts of ABCE1 were detected in Staufen1 RNPs isolated from cells with or without HIV-1 expression (Figure 2A, bottom panel). Several studies have demonstrated that tumor susceptibility gene 101 (TSG101) protein binds the N-terminal p6 region of Gag and is responsible for the release of the virus from the plasma membrane (Sun et al., 1999; Babst et al., 2000; Garrus et al., 2001; VerPlank et al., 2001). We did not find TSG101 in the Staufen1 eluates in any of the cell lines nor could we detect it by mass spectrometry. In fact, other candidate endosomal sorting complex required for transport (ESCRT) proteins were not detected in these Staufen1 RNPs (Miroslav P. Milev and Andrew J. Mouland, data not shown). Thus, despite its interaction with the viral protein Gag, TSG101 appears to be excluded from these particular Staufen1 RNPs suggesting separable functions during HIV-1 replication. Likewise, we did not detect either Vif or Vpr by mass spectrometry and/or western blotting analyses, both well-described interacting partners of Gag (Lavallee et al., 1994; Kondo et al., 1995; Bouyac et al., 1997; Syed and McCrae, 2009). We propose that this negative result could be due to their low abundance in Staufen1 HIV-1 complexes or that these viral proteins interact with distinct subpopulations of Gag that exclude Staufen1 (Klein et al., 2007).

### Identification of proteins associated with Staufen1 HIV-1 RNPs

After validating our RNP purification protocol by western blotting, we proceeded to mass spectrometry analysis to identify proteins that associate with Staufen1 in these particles both in the absence or presence of HIV-1. For these experiments, stably expressing TAP cells were mock transfected or transfected with a proviral plasmid expressing HIV-1 (pNL4-3), lysed and then used for affinity purification. In the eluates from the control TAP samples [Figure 2B, lines (*II*) and (*III*)] we detected a few discrete bands. We sectioned the TAP gel lanes into four pieces (shown with numbers) and analyzed them by mass spectrometry. The proteins that were detected included keratins, immunoglobulins, interferon alpha inducible protein (IFI6), tubulin beta-2 and heat shock protein 90 and since they were found in the control TAP samples, they were considered to be contaminants.

Stable St-TAP cell lines were then mock transfected or transfected with pNL4-3. Lysates were harvested and following SDS-PAGE and Coomassie Blue staining, we observed a similarity in banding pattern between Staufen1-TAP and Staufen1-TAP HIV-1 RNPs [Figure 2B, lines (*IV*) and (*V*)]. In a typical experiment, we excised at least 23 bands from the St-TAP lane and corresponding bands from St-TAP HIV-1 lane. Each band was subjected to LC-MS/MS analysis and the data was concatenated and searched against either the NCBI nr human or virus databases as described in Materials and Methods. A separate randomized decoy database search was performed and the search results were filtered to achieve a False Discovery Rate (FDR) of less than 1%. This corresponded to a score cutoff of 48 and 47 for the human and viral databases, respectively.

**Figure 3:**
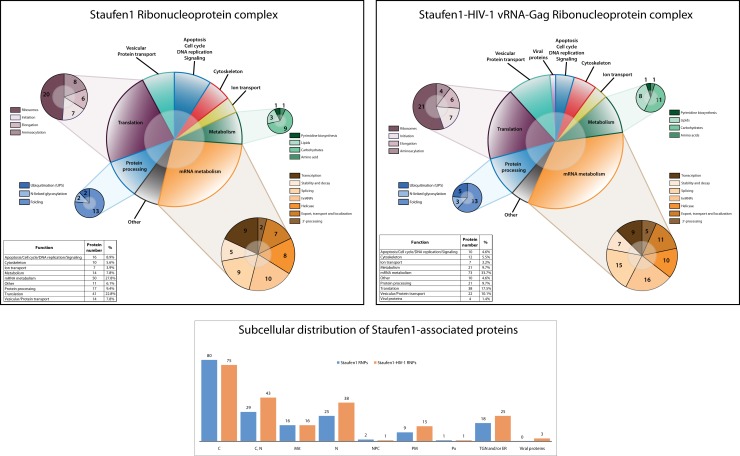
**Staufen1 RNPs contain proteins involved in the localization, stabilization and trafficking of mRNAs and HIV-1 vRNA**. Graphical representation and comparison of Staufen1-associated proteins isolated from 293T cells in the absence (top panel) and the presence of HIV-1 (middle panel). The newly identified proteins were grouped on the basis of their main function or the process in which they are involved in both control and HIV-1 conditions. The subcellular distribution of proteins found in Staufen1-TAP (blue bars) and Staufen1-TAP HIV-1 (orange bars) RNPs is depicted (bottom panel). The information for each protein was obtained from the UniProt database.

We typically detected about 200 proteins in both Staufen1-TAP control and Staufen1-TAP HIV-1 RNPs (Figure 3; Tables S1 and S2 in Supplementary Material). As expected, some of the proteins identified had been previously shown to interact and/or associate with Staufen1, such as hnRNP U, RHA, NFAR, nucleolin, α-tubulin and numerous ribosome subunits (Brendel et al., 2004; Villace et al., 2004). The regulator of nonsense transcripts, Upf1, was also identified (Kim et al., 2005). The majority of the proteins were detected with two or more unique peptides; single peptide hits are also reported in the appended Tables S1 and S2 in Supplementary Material.

We divided all identified proteins from both cohorts into several main categories in respect to their known functions. As shown in the Figure 3, we generated 10 functional categories, including viral proteins in the case when HIV-1 was expressed. The largest group encompassed mRNA-binding proteins that participate in different aspects of mRNA metabolism in the cell (RNA transcription, splicing, stability, transport and degradation) and represent ∼29% of the total number of proteins in the Staufen1 RNPs and ∼35% of the total number of proteins in Staufen1-HIV-1 RNPs. These proteins included numerous heterogeneous nuclear ribonucleoproteins (RNP), DEAD-box family helicases and NMD factors Upf1 and Upf3b [detected by western blot in both types RNPs (Figure 2A) and by mass spectrometry – only in Staufen1 HIV-1 RNPs (Table 1)]. Splicing and mRNA transport factors, such as SFPQ, SF3B2 and endogenous Staufen1 and nucleocytoplasmic shuttling proteins, such as nucleolin, nuclear factor associated with dsRNA, NFAR-1 90 kDa isoform and YB-1 (a universal component of cytoplasmic mRNPs) were also included with this group. Several new mRNA-binding components associated within Staufen1 in both control and HIV-1 conditions. These included leucine-rich protein 130 kDa, LRP130, an RNA-binding protein that accumulates with mRNPs at the nuclear envelope and endoplasmic reticulum (Tsuchiya et al., 2004). Three members of the highly conserved VICKZ family of RNA-binding proteins (Vg1 RBP/Vera, IMP1,2,3, CRD-BP, KOC, ZBP-1) and insulin-like growth factor II mRNA-binding protein 1, 2 and 3 (IMP1 and IMP2 in the native complexes and IMP1 and IMP3 in those purified from HIV-1-expressing cells) were also detected [Reviewed in (Yisraeli, 2005)]. The RNA/DNA-binding protein TDP-43 was detected by mass spectrometry only in HIV-1-containing complexes, but was later confirmed by western blotting analysis in both the presence and absence of HIV-1. Structurally analogous to the hnRNPs, this protein was originally described as a factor that modulates HIV-1 gene expression at the transcriptional level (Ou et al., 1995).

**Table 1:** **Unique proteins identified by mass spectrometry in Staufen1-HIV-1 RNPs**.

Protein name*	UniProt accession number**	Role in HIV-1 replication?	Reference
26S protease regulatory subunit S10B (PSMC6)	P62333	Unknown	N/A
26S proteasome non-ATPase regulatory subunit 2	Q13200	Unknown	N/A
60S ribosomal protein L29 (RPL29)	P47914	Unknown	N/A
7-dehydrocholesterol reductase (DHCR7)	Q9UBM7	Yes	(van’t Wout et al., 2005)
Actin-related protein 2/3 complex subunit 4	P59998	Yes	(Komano et al., 2004; Chertova et al., 2006)
ADP-ribosylation factor 4 (ARF4/ARF2)	P18085	Unknown	N/A
ADP-ribosylation factor 6 (ARF6)	P62330	Yes	(Ono et al., 2004)
Alpha-internexin (INA)	Q16352	Unknown	N/A
AP-2 complex subunit mu (AP-2M1)	Q96CW1	Yes	(Le Gall, 1998; Craig et al., 2000; Batonick et al., 2005)
AP-3 complex subunit delta-1 (AP-3D1)	O14617	Yes	(Dong et al., 2005)
CDP-diacylglycerol–inositol 3-phosphatidyltransferase (CDIPT)	O14735	Unknown	N/A
Cleavage and polyadenylation specificity factor subunit 1 (CPSF1)	Q10570	Unknown	N/A
Cleavage and polyadenylation specificity factor subunit 7 (CPSF7)	Q8N684	Unknown	N/A
Coatomer subunit zeta-1 (COPZ1)	P61923	Unknown	N/A
Copine-3	O75131	Unknown	(Chertova et al., 2006)
Delta-1-pyrroline-5-carboxylate synthetase (ALDH18A1)	P54886	Unknown	N/A
Double-stranded RNA-specific adenosine deaminase (ADAR)	P55265	Yes	(Phuphuakrat et al., 2008; Doria et al., 2009)
Dynamin-2 (DNM2)	P50570	Yes	(Pizzato et al., 2007)
E3 ubiquitin-protein ligase (BRE1A)	Q5VTR2	Unknown	N/A
Env	P03377	Yes	(Freed, 2001)
Eukaryotic initiation factor 4A-III (eIF4A3)	P38919	Unknown	N/A
Eukaryotic translation initiation factor 3 subunit E (eIF3E)	P60228	Unknown	N/A
Gag	P12493	Yes	(Freed, 1998)
Gag/Pol	P12493	Yes	(Jacks et al., 1988)
GTP-binding nuclear protein Ran	P62826	Yes	(Askjaer et al., 1998)
Heat shock 70 kDa protein 1L	P34931	Yes	(Rasheed et al., 2008)
Heterogeneous nuclear ribonucleoprotein A/B	Q99729	Yes	(Mouland et al., 2001)
Heterogeneous nuclear ribonucleoprotein F (hnRNP F)	P52597	Unknown	N/A
Heterogeneous nuclear ribonucleoprotein H2	P55795	Unknown	N/A
Heterogeneous nuclear ribonucleoprotein H3 (hnRNP H3)	P31942	Unknown	N/A
Heterogeneous nuclear ribonucleoprotein Q (hnRNP Q)	O60506	Yes	(Hadian et al., 2009)
Heterogeneous nuclear ribonucleoprotein R (hnRNP R)	O43390	Yes	(Hadian et al., 2009)
HIV-1 Rev-binding protein 2	Q13601	Unknown	N/A
Importin-7	O95373	Yes	(Fassati et al., 2003; Zaitseva et al., 2009)
Insulin-like growth factor II mRNA-binding protein-3	O00425	Unknown	N/A
IQ motif containing GTPase activating protein 1	P46940	Unknown	(Chertova et al., 2006)
Leucine-rich repeat-containing protein 59 (LRRC59)	Q96AG4	Unknown	N/A
Long-chain-fatty-acid–CoA ligase 3 (ACSL3)	O95573	Unknown	N/A
Mannosyl-oligosaccharide glucosidase (MOGS)	Q13724	Unknown	N/A
Nef	P05855	Yes	(Arhel and Kirchhoff, 2009)
NF-kappaB repressing factor (NRF)	A3F768	Yes	(Dreikhausen et al., 2005)
Non-POU domain-containing octamer-binding protein (NONO)	Q15233	Yes	(Zolotukhin et al., 2003)
Nuclear pore complex protein 155 (Nup155)	O75694	Yes	(Brass et al., 2008; Lee et al., 2010)
Peroxiredoxin-6	P30041	Unknown	(Chertova et al., 2006)
Phosphatidylserine synthase 1 (PTDSS1)	P48651	Unknown	N/A
Pre-mRNA 3′-end-processing factor FIP1 (FIP1L1)	Q6UN15	Unknown	N/A
Probable ATP-dependent RNA helicase (DDX17)	Q92841	Unknown	N/A***
Probable ATP-dependent RNA helicase (DDX27)	Q96GQ7	Unknown	N/A
Programmed cell death 8 (AIFM1)	O95831	Unknown	N/A
Protein transport protein Sec61 subunit alpha isoform 1 (SEC61A1)	P61619	Unknown	N/A
Protein tyrosine phosphatase-like protein (PTPLAD1)	Q9P035	Unknown	N/A
Putative RNA-binding protein Luc7-like 2	Q9Y383	Unknown	N/A
Pyruvate dehydrogenase E1 component subunit beta (PDHB)	P11177	Unknown	(Ringrose et al., 2008)
Ras-related GTP-binding protein A (RRAGA)	Q7L523	Unknown	N/A
Ras-related protein Rab-10	P61026	Unknown	(Chertova et al., 2006)
Ras-related protein Rab-5C	P51148	Yes	(Vidricaire and Tremblay, 2005; Chertova et al., 2006)
Ras-related protein Rab-8A	P61006	Unknown	(Chertova et al., 2006)
Ribonucleoprotein PTB-binding 1	Q8IY67	Unknown	N/A
Signal recognition particle receptor subunit beta (SRPRB)	Q9Y5M8	Unknown	N/A
Spliceosome RNA helicase (BAT1)	Q13838	Unknown	(Limou et al., 2009)
Splicing factor, arginine/serine-rich 13A (SFRS13A)	O75494	Unknown	N/A
Splicing factor, arginine/serine-rich 4 (SFRS4)	Q08170	Unknown	N/A
Splicing factor, proline- and glutamine-rich (SFPQ)	P23246	Yes	(Zolotukhin et al., 2003)
T-complex protein 1 subunit beta	P78371	Unknown	N/A
THO complex 4 (THOC4)	Q86V81	Unknown	N/A
THO complex subunit 2 (THOC2)	Q8NI27	Unknown	N/A
T-plastin polypeptide (plastin-3)	P13797	Unknown	N/A
Tubulin alpha-4A chain	P68366	Unknown	(Chertova et al., 2006)
V-type proton ATPase subunit d 1	P61421	Unknown	(Chertova et al., 2006)
Zinc finger RNA-binding protein (ZFR)	Q96KR1	Unknown	N/A

*Viral proteins are highlighted; **Universal Protein Resource — UniProt database [http://www.uniprot.org/]; *** Not applicable.

The second most predominant category relates to proteins involved in RNA translation (∼23 and ∼18% in the absence and presence of HIV-1, respectively) and includes ribosomal, translation initiation and elongation factors and several aminoacyl-tRNA synthetases. Proteins such as PABP1, eukaryotic translation initiation, and elongation factors – eIF3 (α, β and ε), eIF4A (two isoforms – 1 and 3) and eF1 (α, γ and δ isoforms) were also detected.

Proteins involved in cell metabolism represented ∼8% of the total number of proteins in Staufen1-containing RNPs isolated in the absence of HIV-1 and ∼9% in the presence of HIV-1. These included enzymes that regulate different aspects of the general cellular metabolism of carbohydrates, lipids, amino acids, and nucleotides, such as pyruvate kinase and pyruvate dehydrogenase, lactate dehydrogenase and glyceraldehyde-3-phosphate dehydrogenase, fatty acid synthase, ATP-citrate synthase and others (Figure 3; Tables S1 and S2 in Supplementary Material).

Diverse proteins involved in cytoskeleton formation and structure (∼4 and ∼5% in the absence HIV-1 and presence of HIV-1, respectively), including actin, tubulin, vimentin, as well as IQGAP1 were detected (Figure 3; Tables S1 and S2 in Supplementary Material). We also placed Matrin 3 in this category as it is a novel Staufen1-binding partner that was originally reported to be one of the major structural proteins of the inner nuclear matrix (Belgrader et al., 1991). An interesting feature of this protein is its ability to retain A–I edited dsRNAs in the nucleus (Reviewed in DeCerbo and Carmichael, 2005). Moreover, it has been shown in association with APOBEC3G RNPs (Kozak et al., 2006) and with 3′-untranslated region (UTR) of the hepatitis C genome (Harris et al., 2006).

Approximately 8% (−HIV-1) and 10% (+HIV-1) of Staufen1-binding partners were vesicular and protein transport proteins. As mentioned earlier, TSG101, a member of ESCRT-I was not detected by western blotting or by mass spectrometry. Instead, we identified some other proteins involved in the control of endosomal dynamics and in intra-Golgi vesicular transport, including vesicle budding from Golgi membranes. These included Ras-related proteins (Rab-5C, Rab8, Rab-10), some of the coatomer protein complex subunits – COP (α, γ and ζ), adaptor proteins – (AP-2, AP-3) and ADP-ribosylation factors (1, 4, 5 and 6). In addition, some cellular factors regulating the processes of protein folding (heat shock proteins, T-complex proteins, Calnexin), ubiquitination and N-linked glycosylation (Ribophorin-1) constitute the protein processing group [∼8% (−HIV) and ∼9% (+HIV-1)].

Finally, the remaining identified proteins included those involved in ion transport (including sodium/potassium-transporting ATPase, several ATP synthases) and apoptosis/cell cycle/DNA replication/signaling (CDC5L, MCM7 and RACK1), while the remaining proteins were grouped under “others” and included mitochondrial and nucleocytoplasmic transport proteins (Nup155, Importin1, Importin-7, Xpo1, Xpo2 and ADP/ATP translocase; Figure 3 and see Tables S1 and S2 in Supplementary Material).

We detected 45 proteins from Staufen1 HIV-1 RNPs (including viral proteins such as Gag, Gag/Pol, Env and Nef; Tables S1 and S2 in Supplementary Material) that are virion-associated (Ott, 2002, 2008; Cantin et al., 2005; Komano et al., 2005; Chertova et al., 2006; Goff, 2007) representing 22% of the total number of proteins found in the Staufen1 HIV-1 complexes. Among them were RHA, IQGAP1, actin, vimentin, eF1α, Staufen1, IMP1 and heat shock proteins Hsp60, Hsp70 and Hsc70. The latter three are incorporated within the membrane of the viruses and are important for virus infectivity (Gurer et al., 2002). Upf1, which is found in the HIV-1 RNP, also represents a virion-incorporated protein (Abrahamyan et al., 2010).

### Characterization of interactions between Staufen1 and several novel partners using biochemical and immunofluorescence methods

**Figure 4:**
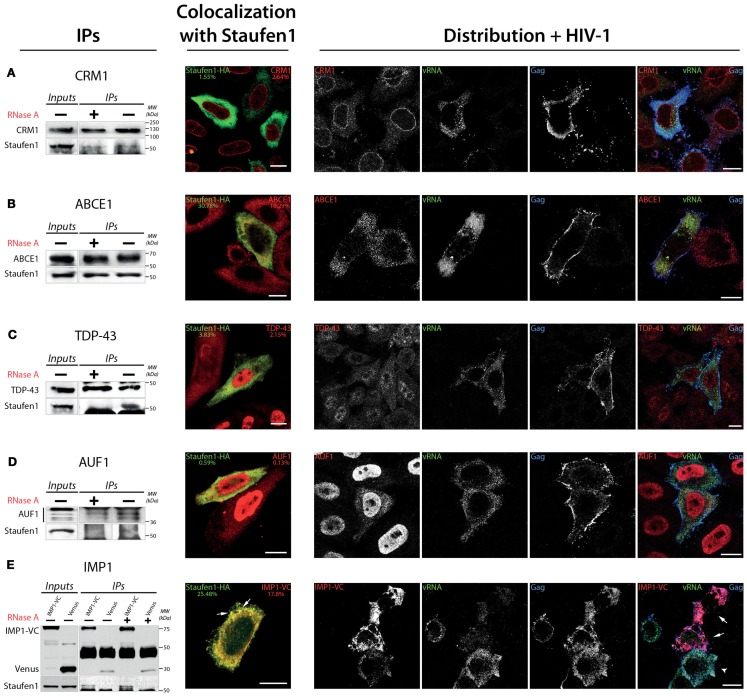
**Confirmation of the *in vivo* association of Staufen1 with several of the host factors that were identified in Staufen1-contain RNP complexes**. **(A–E)** CRM1, ABCE1, TDP-43, AUF1 and IMP1 (as IMP1-VenusC) were immunoprecipitated from 500 mg total protein lysate of HeLa cells. For the immunoprecipitation of IMP1 HeLa cells were transfected with IMP1-VenC or Venus-full length (as a control) and immunoprecipitated with a mouse anti-GFP antibody. The images to the immediate right of the IPs show the patterns of co-localization of CRM1, cytoplasmic RNAse L inhibitor (ABCE1), AUF1, TAR DNA-binding protein (TDP-43) and IMP1 with Staufen1-HA. The Manders’ coefficients (average from >10 cells per experiment derived from Staufen-HA expressing cells only, in %) are shown to provide an estimate of the co-localization. At the far right, the distribution of each host protein (in red) is shown in relation to Gag (blue) and the viral genomic RNA (vRNA, green) in HIV-1-expressing cells as determined by laser scanning confocal microscopy. Cells overexpressing IMP1-VenusC are indicated with white arrows in **(E)**. Size bars are 10 μm.

We confirmed the association of several proteins with Staufen1 complexes using IP and IF analyses. For the purpose of these experiments, we chose three predominantly nuclear proteins: AU-rich element RNA binding protein 1, AUF1, TAR DNA-binding protein, TDP-43 and chromosomal regional maintenance protein 1 (CRM1 or Xpo1), a factor that mediates the nuclear export and ABCE1 (also known an HP68). IP experiments were performed with lysates derived from HeLa cells. To determine the RNA dependence of these interactions, equal amounts of lysates were treated with or without RnaseA for 30 min on ice before IP. Our results demonstrate the RNA-independent character of Staufen1 interactions with CRM1 and ABCE1 (Figures 4A,B, IP panels), whereas those between both Staufen1 and TDP-43 and Staufen1 and AUF1 appeared to be RNA-dependent (Figures 4C,D, IP panels). We detected IMP1 in three independent MS analyses and further confirmed its presence in affinity-isolated complexes using polyclonal rabbit anti-IMP1 antibody (Figure 2A, bottom panel). IMP1 is a human ortholog of chicken Zipcode binding protein 1 (ZBP-1) and belongs to VICKZ protein family (Yisraeli, 2005). Different studies indicate similar functions for IMP1 and Staufen1 with respect to mRNA transport, translational control and localization. IMP1 binds fragile × mental retardation protein (Rackham and Brown, 2004) and PABP1 (Patel and Bag, 2006) and associates with APOBEC3G (Kozak et al., 2006), YB-1, nucleolin and hnRNP A1 (Jonson et al., 2007), proteins that also associate with Staufen1. Recently, IMP1 has been found to bind to HIV-1 Gag (Roy et al., 2006) and in our own work this protein also associates with lipid raft domains (Milev et al., 2010). We performed IP experiments for IMP1 (Figure 4E, IP panel) and observed that its association with Staufen1 was also RNA-independent.

To further demonstrate the interrelationships between Staufen1 and these proteins, we performed laser scanning confocal microscopy to examine their distribution in cells. We transfected HeLa cells with plasmids expressing Staufen1-HA and 24 h later, we fixed and stained the cells with antibodies recognizing the HA-epitope and the endogenous proteins that were used in the IPs, with the exception that we used an anti-GFP to detect IMP1-VenusC (IMP1-VC). We observed co-localization of Staufen1 with ABCE1 and partial co-localization with TDP-43, AUF1 (Lund et al., 2012) and CRM1 [Figures 4A–D, co-localization with Staufen1 panels; Manders’ coefficients (%) are shown]. As expected, Staufen1 and IMP1 co-localized in cytoplasmic particles, but this was only true for a proportion of these proteins (Figure 4E, *co-localization with Staufen1 panel*). Finally, the possible interactions of these novel Staufen1 partners with vRNA and Gag were elucidated by combined FISH and IF co-analyses in HIV-1-expressing cells. We transfected HeLa cells with both pNL4-3 proviral DNA plasmids coding for IMP1-VenusC (Figures 4A–E, *distribution *+ *HIV-1 panels*). Cells were fixed and stained with rabbit anti-CRM1, anti-ABCE1 anti-TDP-43, anti-AUF1, or anti-GFP (to detect IMP1-VenusC; all in red, Figures 4A–E, *distribution *+ *HIV-1 panels*), along with sheep anti-p17 to detect Gag (shown in blue) and FISH was performed to detect viral RNA (shown in green). The presence of HIV-1 caused the partial accumulation of AUF1 in the cytoplasm. The mechanism, however, that underlines this phenomenon is unclear but could be due to the HIV-1 imposed block on nuclear import (Monette et al., 2009) and the functional significance of this interaction was recently demonstrated (Lund et al., 2012). Importantly, ABCE1, currently implicated in the generation of HIV-1 capsids, co-localized with the vRNA. At present, we do not know if this is via a direct or indirect interaction. The co-localization of IMP1 with viral components was estimated in cells simultaneously transfected with IMP1-VenusC and pNL4-3 plasmids. We noticed that cells overexpressing IMP1-VenusC (Figure 4E, shown with white arrows) had an apparent decrease in the signal for vRNA. Cells that did not express or expressed IMP1-VenusC at lower levels showed higher accumulations of vRNA (Figure 4E, white arrow head). This negative effect on vRNA in HIV-1 positive cells was observed in more than 55% of the cells.

### Sucrose density gradient analysis of Staufen1 HIV-1 RNPs

**Figure 5:**
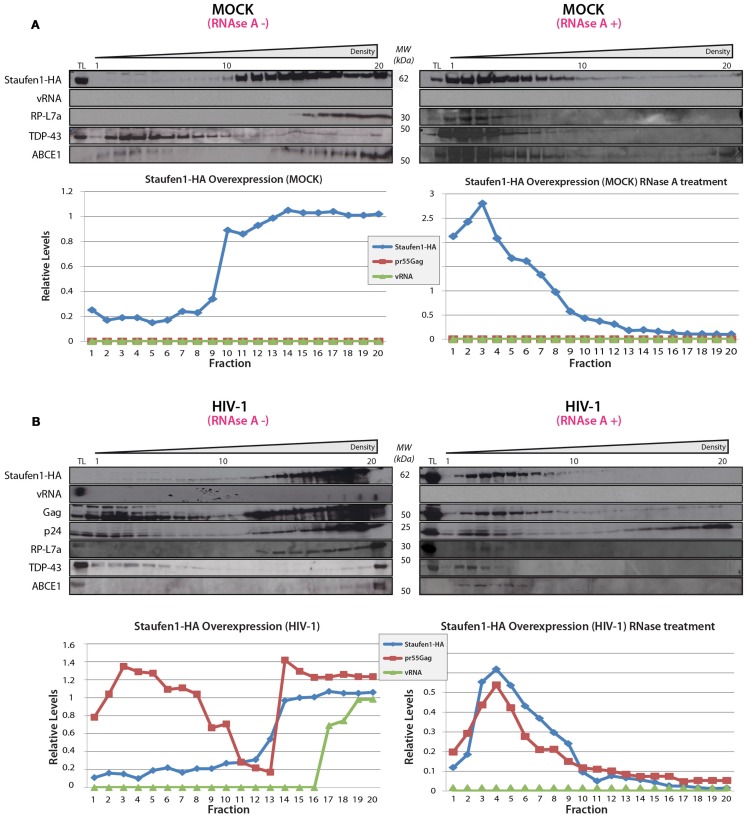
**Staufen1 co-fractionates with Gag and vRNA in gradient density fractionation analyses**. **(A)** HeLa cells were either mock transfected with empty vector, pcDNA3, or with a plasmid that expresses Staufen1-HA. The transfected cells were collected 24 h later, lysed and were either mock-treated or treated with RNAse A. The lysates were then fractionated on 5–50% sucrose gradients and 20 fractions were collected for further analysis by western blotting for viral and host proteins, as indicated. HIV-1 viral genomic RNA (vRNA, 9 kb) was assessed in each fraction by slot blot analysis. TL represents the total lysates. **(B)** HeLa cells were co-transfected with pNL 4–3 and Staufen1-HA. The presence of Staufen1-HA, precursor Gag and p24 were assessed in each fraction by western blotting analysis. HIV-1 viral genomic RNA (9 kb, vRNA) was assessed in each fraction by slot blot analysis. Staufen1, Gag and vRNA were quantitated in each fraction by densitometry and relative levels are depicted for each fraction (Blue: Staufen1-HA, Red: Gag, Green-vRNA).

**Figure 6:**
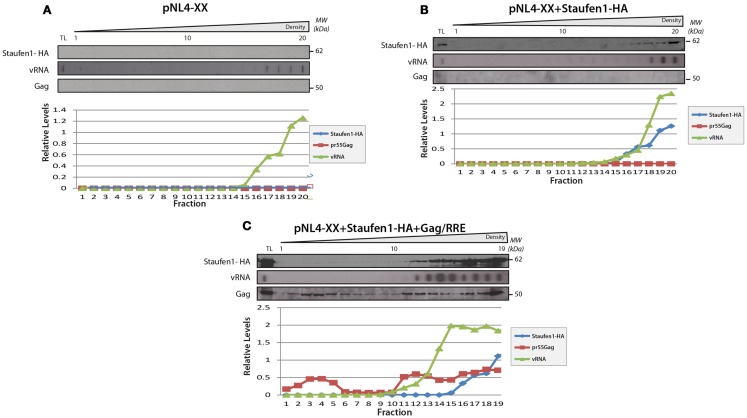
**The sedimentation of Staufen1 and vRNA indensitygradientsdid not significantly change in the absence of Gag expression**. **(A)** HeLa cells were co-transfected with pNL4-XX (harboring two mutations in the *gag* open reading frame to prevent Gag synthesis) with either empty vector control **(A)**, Staufen1-HA **(B)**, or Gag-RRE **(C)**. The cells lysates were then fractionated on 5–50% sucrose gradients and 20 fractions were collected for further analysis by western blotting analyses, as indicated. TL represents the total lysates. Staufen1, Gag and vRNA were quantitated in each fraction by densitometry and relative levels are depicted for each fraction (Blue: Staufen1-HA, Red: Gag, Green: vRNA).

Sucrose density gradient analyses were then performed to further characterize Staufen1 RNP dynamics when HIV-1 was expressed. The biochemical examination of these RNPs is important since Staufen1 is found in several RNPs relevant to HIV-1 (Chiu et al., 2006; Abrahamyan et al., 2010) but it also selectively associates with the vRNA in complex with Gag and other host proteins (Chatel-Chaix et al., 2004, 2008; Ajamian et al., 2008). Moreover, in our earlier study, we demonstrated that Staufen1 RNPs were plastic in size but co-associated with Gag and vRNA signals (Abrahamyan et al., 2010). We therefore assessed the distribution of Staufen1 RNPs by sucrose density centrifugation to determine how HIV-1 influences this. HA-Staufen1 was transfected into HeLa cells without or with pNL4-3 proviral HIV-1 DNA. Lysates were processed for sucrose density gradient analyses. Fractions were collected from the top of the gradient and HIV-1 vRNA was quantitated by slot blot analysis. An aliquot of each gradient fraction was run on SDS-PAGE and western blotting was performed to identify Staufen1-HA and several of the newly identified members of Staufen1-TAP-containing host proteins (e.g., RP-L7a, TDP-43 and ABCE1, Table S2 in Supplementary Material) and Gag in HIV-1 samples (Figures 5A,B). In some cases, lysates were treated with RNAse A to determine RNA-dependency. These analyses revealed that there was no consistent shift in the sedimentation profile of Staufen1-HA complexes in mock and HIV-1-expressing cells and they mainly fractionated in denser fractions #11–18 (Figure 5A). This was also true for those for the selected Staufen1-associated host proteins. However, Gag sedimented in two regions of the density gradient, in both a light (fractions #1–7) and a more dense region (fractions #12–20; Figure 5B), while the vRNA only sedimented in very dense fractions (#16–20) in this assay. RNAse A treatment eliminated the vRNA, but also disrupted the distribution of viral and host proteins in the dense fractions leading to their migration in lighter density fractions, with the exception of the processed form of Gag, p24, which likely represents capsids or virus particles that are membrane-bound (Levesque et al., 2006). The precursor to p24, p25 was also observed in the lighter fractions supporting this notion (Figure 5 and below). We have shown that the Staufen1-Gag interaction is RNA-independent and in close proximity (Chatel-Chaix et al., 2004) so these results indicate that Gag is recruited to pre-existing Staufen1 RNPs as we demonstrated earlier (Milev et al., 2010) but also, it remains associated to Staufen1 when viral and cellular RNAs are in limiting supply. This notion is also supported in experiments in which a Gag-less pNL4-3 is expressed. This proviral DNA will express all viral proteins and RNAs except for Gag and Gag/Pol (Poon et al., 2002). When expressed alone (Figure 6A) or when Staufen1-HA is overexpressed (Figure 6B), the sedimentation profiles for vRNA were not appreciably affected in the absence of Gag. This is consistent with our recent findings that showed that Staufen1 and vRNA co-localized significantly in the absence of Gag under the same experimental conditions (Abrahamyan et al., 2010). When Gag expression was rescued by the expression of a Rev-dependent Gag expressor (Lingappa et al., 2006; Figure 6C), the distribution of vRNA was consistently found to be shifted toward the lighter fractions, that might be specific to this rescue experiment (i.e., due to *trans* expression of Gag) because the vRNA is found in the penultimate fractions when pNL4-3 is expressed (Figure 5). Further experimentation will be required to characterize the function of these lighter RNPs upon Gag rescue in *trans*.

**Figure 7:**
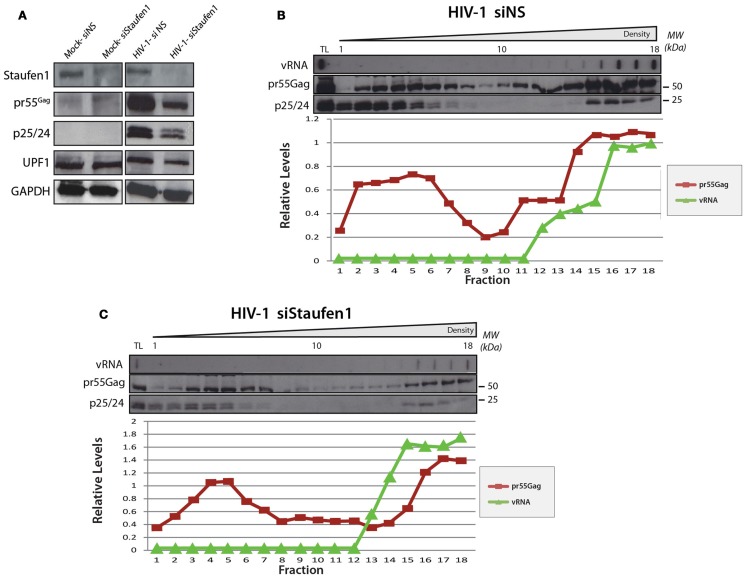
**The relative levels of Gag in light and dense gradient fractions are modulatedin Staufen1-depleted cells**. HeLa cells were mock transfected or co-transfected with pNL4-3 with either non-silencing control siRNA (siNS) or a siRNA to deplete endogenous Staufen1 (siStaufen1) and were harvested and lysed 36 h later and analyzed by western blotting. Corresponding expression levels for Staufen1, Gag, UPF1 and GAPDH proteins are shown in **(A)**. The cell lysates from siNS-treated **(B)** and siStaufen1-treated **(C)** cells were fractionated on 5–50% sucrose gradients and 19–20 fractions were collected for further analysis by western blotting, as indicated. TL represents the total lysates. HIV-1 viral genomic RNA (9 kb, vRNA) was assessed in each fraction by slot blot analysis. Staufen1, Gag and vRNA were quantitated in each fraction by densitometry and relative levels are depicted for each fraction (Blue: Staufen1-HA, Red: Gag, Green: vRNA).

In the experiments above, endogenous Staufen1 is abundantly expressed that could allow the assembly of Staufen1 viral RNPs. In conditions when endogenous Staufen1 is depleted by siRNA, we have shown that large Staufen1 RNPs (SHRNPs) formed in the vicinity of the nucleus and in the cytoplasm. These structures were proposed to be supraphysiologic RNPs that could serve as scaffolds for viral assembly and vRNA encapsidation. We investigated by density gradient analysis whether the SHRNPs represented a super-dense RNP or amalgamations of many smaller RNPs. HeLa cells were transfected with pNL4-3 DNA and either siNS or siStaufen1 siRNAs and lysates were prepared for gradient analyses. While siStaufen1 treatment again resulted in reduced Gag synthesis (Abrahamyan et al., 2010; Figure 7A), the sedimentation profiles for vRNA and Gag did not appreciably change (Figures 7B,C). However, the relative levels of Gag in light and dense gradient fractions were modulated such that a less abundant signal for Gag in the dense fractions was observed. This could be the populations of Gag influenced by Staufen1 expression levels and functionally relevant for the assembly of dense RNPs or represent a population of Gag with a specific function (Klein et al., 2007). Importantly, these results provide evidence that the larger Staufen1 RNPs observed in Staufen1 depletion conditions likely represent aggregations of similar-sized RNPs rather than the assembly of a supraphysiologic RNP (Abrahamyan et al., 2010).

## Discussion

The combination of TAP and mass spectrometry has proven to be reliable for the characterization of different protein complexes (reviewed in Xu et al., 2010) including those containing HIV-1 Gag, UPF1 and Staufen1 (Schell et al., 2003; Brendel et al., 2004; Villace et al., 2004; Roy et al., 2006). Here we provide deeper insights into the impact of HIV-1 expression on the composition of Staufen1 RNPs.

While the analyses performed here detected almost all of the previously characterized proteins characterized as components of Staufen1 RNPs (Brendel et al., 2004; Villace et al., 2004), they also revealed that the protein composition of the particles was influenced by the presence of HIV-1. In addition to the viral gene products, Gag, Gag/Pol, Env and Nef, which are all incorporated into Staufen1 RNPs in the presence of HIV-1, generally the number of proteins in the Staufen1 RNP was enhanced in the presence of HIV-1 (Figure 3; Table 1; Tables S1 and S2 in Supplementary Material). In addition, HIV-1-mediated an increase in the amounts of several other host proteins that were found in smaller amounts in Staufen1-containing RNPs isolated from uninfected 293T cells (Figure 2A). The signal intensities of UPF1, eF1α, ribosomal protein L7a and RHA, for example, were approximately threefold greater in western blotting experiments in the presence of HIV-1. Endogenous Staufen1 was also recruited to a higher degree to the complexes isolated from HIV-1-expressing cells (Figure 2A, top panels). This HIV-1-mediated modulation in protein composition of the Staufen1 RNA was indeed selective since equal quantities of several other proteins, such as IMP1, ABCE1 and UPF3 were found between the two treatment groups. The changes to the Staufen1 protein environment described here could invariably be mediated by the presence of the four viral proteins (Gag, Gag/Pol, Env and Nef) and the vRNA within the Staufen1 HIV-1 RNP, thereby recruiting and enriching for a number of host factors. Consistently, Gag can recruit Staufen1 and other host factors (Milev et al., 2010). When this analysis was performed in Jurkat T cell lines, we did not observe some of the same changes to the abundance of proteins (e.g., TDP-43) as judged in western blots (Figure 2C). This could possibly be due to lower transfection efficiencies, or more likely, because the compactness of the cytoplasm in Jurkat T cells and the relatively shorter intracellular distances in these mononuclear cells might restrict the protein content of Staufen1 RNPs in general. Overall, it will be important to analyze further the possible functions of the unique proteins found in Staufen1 HIV-1 RNPs and their effects on HIV-1 replication.

### Constituents of Staufen1 RNPs involved in mRNA translation, trafficking and stability

We identified numerous Staufen1-associated proteins including the small and large ribosomal subunit proteins, translation initiation and elongation factors, tRNA synthetases, some of which are involved in RNA trafficking RNPs (Carson et al., 2008). Most of these proteins were common components that were identified in both native Staufen1 and Staufen1 HIV-1 RNPs as it was in the cases of eIF3α and eIF4A1 (Tables S1 and S2 in Supplementary Material). However, eIF3β was only found in native and eIF3ε was only detected in Staufen1 HIV-1 RNPs (Table 1; Tables S1 and S2 in Supplementary Material). In the context of the Staufen1 HIV-1 RNPs, their functions in mRNA translation could be potentially enhanced by the presence of the viral proteins and evidence for this is supported in the literature (Jager et al., 2012). For example, Env, influences RPS6 kinase and upregulates mRNA translation (Barcova et al., 1999). Furthermore, HIV-1 Gag and that of other retroviruses can modulate the translation activity of its cognate mRNA (the vRNA; Sonstegard and Hackett, 1996; Anderson and Lever, 2006).

Proteins involved in mRNA stability were also common to both native Staufen1 and Staufen1 HIV-1 RNPs including Upf1, Upf3b, IMP1 and FUSE-binding protein (refer to Tables S1 and S2 in Supplementary Material for others), but IMP2 and IMP3 were exclusive to native and to Staufen1 HIV-1 complexes, respectively. Although several of these factors have already been shown to alter HIV-1 gene expression (Ajamian et al., 2008; Zhou et al., 2008), additional work will be needed to determine their roles in Staufen1-containing RNPs. Nevertheless, the presence of these proteins suggests that Staufen1 RNPs are involved in the regulation of mRNA translation and stability of both cellular and viral mRNAs. Many components identified as constituents of stress granules (Ohn et al., 2008) were also observed in Staufen1 RNPs including Staufen1 itself, several RNA helicases, translation factors (eIF3, eEF1), IMP1 and ribosomal proteins (Table S2 in Supplementary Material) and their presence or sequestration could be implicated in the abrogation of this stress response in HIV-1- and other virus-infected cells (Abrahamyan et al., 2010; Ruggieri et al., 2012).

### Staufen1 RNPs: implications in transcription, splicing and mRNA nuclear retention?

Staufen1 RNP also contain a number of proteins implicated in nuclear RNA quality control, a role we have previously characterized for UPF1 (Ajamian et al., 2008). We detected several predominantly nuclear proteins in native Staufen1 and Staufen1 HIV-1 RNPs such as nucleolin (NCL), hnRNPU and RNA helicase (RHA or DDX9; Brendel et al., 2004; Villace et al., 2004) and several transcription and splicing factors as common elements in both control and HIV-1 Staufen1 RNPs (Tables S1 and S2 in Supplementary Material), but there were also significant differences. Staufen1 HIV-1 RNPs appeared to be enriched in splicing factors, RNA helicases and hnRNPs (refer to Table 1) that are specifically co-opted by HIV-1 and have established roles in HIV-1 replication (Caputi et al., 1999; Jeang and Yedavalli, 2006; Levesque et al., 2006; Lund et al., 2012). The NF-κB repressing factor (NRF), for instance, binds to a specific negative regulatory element within the HIV-1 LTR and regulates transcription initiation and elongation (Dreikhausen et al., 2005). Two other RNA- and DNA-binding proteins SFPQ (PSF) and Non-POU domain-containing octamer-binding protein (NONO) act in the context of a heterodimer and play essential roles in the transcriptional regulation and in the pre-mRNA splicing. As well, both proteins play a role in HIV-1 replication by binding, with high affinity, the instability (INS) regions in HIV-1 *gag* mRNA suggesting a role in RNA stability or translation (Zolotukhin et al., 2003). Importantly, NONO and PSF form a complex with another common Staufen1 component, nuclear matrix protein Matrin 3, as described above (Zhang and Carmichael, 2001; DeCerbo and Carmichael, 2005). Since Staufen1 can shuttle between the nucleus and cytoplasm (Martel et al., 2006), these potential associations represent potential therapeutic targets in the context of the transporting Staufen1 RNP.

### Staufen1 RNPs factors involved in general metabolism and cholesterol biogenesis

Surprisingly and exclusive to Staufen1 HIV-1 RNPs, we detected phosphatidylserine synthase 1 (PTDSS1), long-chain-fatty-acid-CoA ligase 3 (ACSL3) and 7-dehydrocholesterol reductase (DHCR7), the latter catalyzing the generation of cholesterol (Table 1). Gag’s interaction with Staufen1 on cholesterol-rich membranes could suggest a functional implication in cellular cholesterol biogenesis (Milev et al., 2010) in that rerouting cholesterol-rich membranes toward the periphery promotes virus release and infectivity (Liao et al., 2001; Tang et al., 2009; Coleman et al., 2012). Likewise, Nef was also detected in Staufen1 HIV-1 RNPs and this viral protein could also influence assembly and trafficking since it induces cholesterol biosynthesis genes (Table 1; Zheng et al., 2003; van’t Wout et al., 2005). These findings favor the idea that the processes of cholesterol biosynthesis, the formation of lipid rafts and the trafficking of viral components might be associated to Staufen1-containing complexes.

### Staufen1-binding partners involved in vesicular trafficking

We identified multiple proteins that regulate vesicle biogenesis and trafficking within Staufen1 RNPs (±HIV-1), with twice the number of proteins with related functions when HIV-1 was expressed. Several common components were detected and known to be involved in the intracellular vesicular transport between the plasma membrane, endoplasmic reticulum and Golgi membranes including the coatomer subunit alpha (COPA; Beck et al., 2009), vesicle-fusing ATPase (NSF; Zhao et al., 2007) and adaptor protein AP-3S1 (sigma subunit; Tables S1 and S2 in Supplementary Material). The coatomer subunit zeta-1 (COPZ1), ADP-ribosylation factors ARF4 and ARF6, several Rab-related proteins (Rab-5C, -8A and -10), adaptor proteins AP-2M1 (mu subunit), AP-3D1 (delta subunit), which importantly, interact with Nef and Env (i.e., AP-2, mu; Boge et al., 1998; Craig et al., 2000) and Gag (i.e., AP-2, mu; AP-3, delta; Batonick et al., 2005; Dong et al., 2005) all uniquely associated to the Staufen1 HIV-1 RNPs (Table 1). We also identified a putative YXXØ (where Ø is a bulky hydrophobic residue) sorting signal in the dsRBD3 of Staufen1 (similar to that found in both Gag and Env; Batonick et al., 2005) that may mediate the mu subunit binding. Likewise, the existence of several di-leucine sorting motifs in Staufen1 could mediate binding to COP1 beta (Ohno et al., 1995; Rapoport et al., 1998). These observations support the notion that the mRNA/HIV-1 vRNA transport pathways are coupled with organized vesicular trafficking in cells (Cohen, 2005; Baumann et al., 2012), that is strengthened by several recent observations that vRNA traffics on endosomal membranes during HIV-1 egress and assembly (Lehmann et al., 2009; Molle et al., 2009).

Most of the biochemical evidence points to a role for Staufen1 RNPs in vRNA fate. The co-sedimentation of several components with Staufen1, using the available antibodies, reveals co-associations that are likely to be functionally relevant in this context and important for HIV-1-mediated disease (e.g., Tosar et al., 2012). Indeed, several of these associations have been characterized previously, for example those that have been characterized between Gag and ABCE1, Staufen1, UPF1, AUF1, IMP1 and RNA helicases. These RNPs appear to be static in size (Figure 6), but are mobile since they can aggregate in clusters when Staufen1 is in limiting supply (Abrahamyan et al., 2010). The disruption of all of these protein complexes upon RNAse A treatment reveals the dependence on viral and/or cellular RNA but also indicates that the targeting of RNA-related phenomena will be a suitable therapeutic approach in the future (Radi et al., 2012).

## Conclusion

Staufen1 has multiple roles during HIV-1 replication including one in Gag multimerization and another in genomic RNA encapsidation. The present study applied tandem affinity immuno-purification techniques coupled with mass spectrometry to characterize the composition of Staufen1 RNPs in HIV-1-expressing cells. Our results demonstrate that HIV-1 induces substantial changes to the composition of cellular Staufen1 RNPs. The identification of the associated host and viral factors to the Staufen1 RNP will contribute to a better understanding of how HIV-1 co-opts subcellular RNPs and machineries to achieve intracellular trafficking, efficient gene expression and the correct localization and fate of its genomic RNA.

## Authors’ Contributions

Miroslav P. Milev, Mukunthan Ravichandran and Andrew J. Mouland designed the experiments, analyzed the data and drafted the manuscript; Miroslav P. Milev and Mukunthan Ravichandran performed the experiments; Morgan F. Khan and David C. Schriemer performed LC-MS/MS analyses, peptide and protein identifications and analyzed the mass spectrometry results. All authors edited and approved the final manuscript.

## Conflict of Interest Statement

The authors declare that the research was conducted in the absence of any commercial or financial relationships that could be construed as a potential conflict of interest.

## Supplementary Material

The Supplementary Material (Tables S1 and S2) for this article can be found online at: http://www.frontiersin.org/Virology/10.3389/fmicb.2012.00367/abstract
